# A Comparative Study on the Safety and Efficacy of Erbium Laser Therapy Versus Traditional Treatments in Managing Dentin Hypersensitivity: An Observational Study

**DOI:** 10.7759/cureus.65022

**Published:** 2024-07-21

**Authors:** Sajith S, Vivek H P, Shanvi Ray, Praveena Sharma S, Abighana Mannepalli, Mohammad Ismail

**Affiliations:** 1 Department of Conservative Dentistry and Endodontics, Coorg Institute of Dental Sciences, Coorg, IND; 2 Department of Public Health Dentistry, College of Dental Sciences, Davangere, IND; 3 Department of Periodontology, Align Dental Clinic, New Delhi, IND; 4 Department of Conservative Dentistry and Endodontics, College of Dental Sciences, Davangere, IND; 5 Department of Periodontology, Navodaya Dental College, Raichur, IND; 6 Department of Periodontology, Mithila Minority Dental College and Hospital, Darbhanga, IND

**Keywords:** dental interventions, visual analog scale, topical fluorides, laser therapy, dentin hypersensitivity

## Abstract

Background

This study aimed to explore the potential efficacy and safety of laser therapy compared with traditional desensitizing treatments in the management of dentin hypersensitivity.

Methodology

A comprehensive observational study was conducted on 138 adult individuals aged 18-65 diagnosed with dentin hypersensitivity. Participants were allocated to either the laser therapy or traditional treatment group. The laser therapy group received treatment using the Fotona LightWalker® Erbium laser at 2,940 nm. The energy density was set at 20 J/cm² using continuous and contact modes, with the laser tip held perpendicularly to the irradiated site. Each session lasted five minutes, conducted bi-weekly for three months. Traditional treatment included the in-office application of 5% sodium fluoride varnish application once every 15 days for three months and the use of desensitizing toothpaste as part of regular oral hygiene routines. Follow-up assessments were conducted 6 and 12 months post-treatment to evaluate the longevity and stability of the treatment effects. Primary outcomes were assessed by dentin hypersensitivity reduction measured using Visual Analog Scale (VAS) scores and tactile hypersensitivity assessments.

Results

Laser therapy consistently surpassed traditional treatment in reducing dentin hypersensitivity, as reflected by the significantly lower VAS scores. Notably, at 3, 6, and 12 months, laser therapy demonstrated mean VAS scores of 2.5 (±1.5), 1.2 (±0.9), and 0.6 (±0.5), respectively, while the traditional treatment group exhibited higher scores (3.8 ± 1.2, 4.5 ± 1.0, and 4.0 ± 0.7, respectively). Statistical analysis revealed that these differences were highly significant (p < 0.001). Tactile hypersensitivity assessments echoed these findings, with laser therapy consistently maintaining lower scores (0.8 ± 0.7 at 6 months, 0.4 ± 0.3 at 12 months) compared to traditional treatment (3.5 ± 1.0 at 6 months, 4.0 ± 0.7 at 12 months) with statistical significance at all time points (p < 0.001).

Conclusions

Although this study lacks a randomized controlled design, the observed substantial reduction in VAS scores and tactile hypersensitivity assessments, along with the favorable safety profile of laser therapy, suggest its potential as an effective alternative for managing dentin hypersensitivity.

## Introduction

Dentin hypersensitivity, characterized by sharp, transient pain stemming from exposed dentin in response to external stimuli, represents a persistent challenge in dental care, affecting a substantial proportion of the global population [1,2]. While conventional desensitizing treatments have been the mainstay, their variable efficacy and limitations necessitate the exploration of alternative modalities to provide effective and enduring relief [3-5]. Among these emerging approaches, laser therapy has garnered attention for its potential to address dentin hypersensitivity through the modulation of neural responses, promotion of dentin tubule occlusion, and influence on dentin permeability [6,7].

Despite promising initial results, the body of evidence supporting laser therapy for dentin hypersensitivity remains nascent, prompting the need for a comprehensive investigation. This study was designed to address this gap by rigorously evaluating and comparing the efficacy and safety of laser therapy with traditional desensitizing treatments. This study aimed to provide essential insights into the potential of laser therapy to offer sustained relief and overcome the limitations of the existing treatments.

The rationale for exploring laser therapy lies in its potential for a novel and precise approach to managing dentin hypersensitivity. Laser therapy utilizes specific wavelengths that interact variably with dental tissues based on their composition, such as water, hydroxyapatite, hemoglobin, and pigmentation [8]. This variability in tissue interaction, governed by the absorption coefficient of the laser, suggests that certain lasers can effectively target specific chromophores within the dental tissues. By examining these interactions, this study aimed to provide an evidence-based assessment of the efficacy and safety of laser therapy compared to traditional desensitizing treatments. Additionally, this study focused on both subjective and objective measures to offer a comprehensive understanding of the impact of these interventions on patients [9].

The significance of this study extends beyond its immediate focus on dentin hypersensitivity. Laser therapy has gained attention in various dental applications, including periodontics and oral surgery, and understanding its efficacy in addressing dentin hypersensitivity contributes to the broader knowledge base on laser applications in dentistry [9-11]. This study may inform dental practitioners about the potential of incorporating laser therapy into their treatment protocols, aligning with the growing demand for patient-friendly interventions.

The primary objective of this study was to assess and compare the efficacy of laser therapy and traditional desensitizing treatments for dentin hypersensitivity. The results of this study are expected to provide valuable insights into the comparative effectiveness of laser therapy and traditional treatment. Furthermore, this study addresses the absence of a null hypothesis by rigorously evaluating and comparing the efficacy and safety of laser therapy with traditional desensitizing treatments. This approach aims to provide essential insights into the potential of laser therapy to offer sustained relief and overcome the limitations of existing treatments.

## Materials and methods

This study was conducted in the Department of Periodontology, adhering to the highest ethical standards outlined in the Declaration of Helsinki, to ensure the welfare, rights, and privacy of all participants throughout the study duration. Ethical approval was obtained from the institutional review board of Coorg Institute of Dental Sciences (approval number: IEC/CIDS/2022/11145). Informed consent was obtained from the participants, and confidentiality was rigorously maintained with anonymized data during the analysis.

Participants

The study enrolled a carefully selected cohort of 138 adult individuals, aged 18-65 years, who were clinically diagnosed with dentin hypersensitivity. Stringent inclusion criteria were applied to ensure the relevance of the study population to the research objective. The exclusion criteria were equally comprehensive, encompassing individuals with contraindications to laser therapy, those with severe periodontal disease, pregnant individuals, and those with any medical conditions that could potentially interfere with their active participation in the study.

Randomization process

To ensure an unbiased and balanced distribution of participants, a robust randomization process was employed. Participants were randomly assigned to either the laser therapy group or the traditional treatment group. This assignment was achieved through the utilization of computer-generated randomization lists, eliminating any potential selection bias, and enhancing the internal validity of the study. The meticulous randomization process aimed to create comparable groups at baseline, thus facilitating a more rigorous and reliable evaluation of the interventions.

Interventions

The laser therapy group received a carefully designed intervention utilizing the Fotona LightWalker® Erbium dental laser system. The laser operated at a wavelength of 2,940 nm, targeting dental tissues because water absorption leads to efficient ablation and minimal thermal damage. However, hydroxyapatite, a major component of tooth structure, also plays a role in laser interaction, albeit to a lesser extent compared to water. However, the energy density was set at 20 J/cm² with continuous and contact modes. The laser tip of the device was perpendicularly held at the irradiation spot. The laser session lasted approximately five minutes, allowing for precise and controlled exposure. Participants underwent treatment sessions bi-weekly for three months.

Before commencing laser therapy, participants received detailed explanations about the entire procedure to ensure their full understanding and cooperation. Potential side effects, such as mild temporary discomfort, were thoroughly communicated to manage participant expectations, with emphasis placed on the transient nature of these effects. A comprehensive discussion on post-treatment care practices was provided, including recommendations for oral hygiene and necessary precautions to optimize treatment efficacy and ensure participant comfort.

Participants assigned to the traditional treatment group underwent a systematic and standardized desensitizing regimen adhering to conventional protocols recognized in clinical practice. The treatments included fluoride application (5% sodium fluoride varnish) and the use of desensitizing toothpaste as part of regular oral hygiene routines. Fluoride application was conducted in the dental office setting once every 15 days for three months. The dental professional applied the fluoride varnish directly to the affected areas of the teeth. Participants were instructed to avoid eating, drinking, or brushing their teeth for at least 30 minutes after the application to ensure maximum fluoride uptake and effectiveness.

Participants were instructed to brush their teeth with the desensitizing toothpaste twice daily (morning and night). Using a pea-sized amount of toothpaste on a soft-bristled toothbrush, patients were advised to brush gently but thoroughly, covering all surfaces of the teeth, especially focusing on areas experiencing sensitivity. After brushing, participants were advised to spit out the excess toothpaste but not to rinse their mouth immediately, allowing the active ingredients more time to work on the tooth surfaces.

Participants’ adherence to the traditional treatment regimen was closely monitored throughout the study. Regular follow-up appointments allowed for assessments of compliance, evaluation of treatment efficacy, and documentation of any adverse events or concerns raised by participants.

Outcome measures

Primary Outcomes

Dentin hypersensitivity reduction was quantified through the utilization of the Visual Analog Scale (VAS), which consists of a long horizontal line, in which the patient pointed out their pain using a pain scale similar to the Faces Pain Scale [11]. This subjective measure allowed participants to rate the intensity of their dentin hypersensitivity on a continuous scale, providing a nuanced and quantitative assessment of their individual experiences. VAS scores served as the primary endpoint to gauge the effectiveness of the interventions.

Tactile hypersensitivity, a pivotal dimension of dentin hypersensitivity, was systematically evaluated. The tactile stimulus was examined using an explorer (#17/23), passing at a right angle to the bucco-cervical tooth surface of concern [11]. This objective assessment, complementing the subjective VAS scores, offered a comprehensive understanding of treatment efficacy by objectively capturing the responsiveness of the affected dentin to tactile stimuli. The combination of subjective and objective measures strengthened the overall assessment of the treatment outcomes.

Secondary Outcomes

A robust system for monitoring adverse events was implemented throughout the study period. The participants’ reports of any untoward events or discomfort were documented. These events were then systematically categorized based on their severity, allowing for a comprehensive analysis of safety outcomes. Any reported adverse events were promptly addressed to ensure the safety and well-being of study participants. This meticulous approach to adverse event monitoring adds an additional layer of insight into the safety profile of the interventions, contributing to a holistic evaluation of the study outcomes. Follow-up assessments were conducted 6 and 12 months post-treatment to evaluate the longevity and stability of the treatment effects.

Data analysis

The collected data underwent rigorous statistical analysis using SPSS Statistics version 22 (IBM Corp., Armonk, NY, USA) which facilitated the application of appropriate statistical tests including t-tests tailored to the nature of the data. Statistical findings were interpreted at a predetermined significance level of p < 0.05.

## Results

The participant demographic data in Table 1 illustrate a well-balanced distribution between the laser therapy and traditional treatment groups, both in terms of age and sex.

**Table 1:** Participant demographics. SD: standard deviation; n: number of participants

Characteristic	Laser therapy group (n = 69)	Traditional treatment group (n = 69)	Total (N = 138)
Mean age ± SD (years)	42.5 ± 8.2	41.8 ± 7.5	42.1 ± 7.9
Gender			
Male	30	32	62
Female	39	37	76

Participants in the laser therapy group had a mean age of 42.5 (±8.2) years, while those in the traditional treatment group had a mean age of 41.8 (±7.5) years. The sex distribution was also comparable, with 30 males and 39 females in the laser therapy group and 32 males and 37 females in the traditional treatment group. This demographic balance enhances the internal validity of the study, ensuring that any observed differences in outcomes can be attributed to interventions rather than to demographic variations.

As shown in Table 2, which presents the primary outcome of dentin hypersensitivity reduction using VAS scores, the baseline VAS scores were 7.8 (±1.2) for the laser therapy group and 7.6 (±1.1) for the traditional group.

**Table 2:** Visual Analog Scale (VAS) scores. A p-value <0.05 was considered significant. SD: standard deviation

Time point	Laser therapy group (mean ± SD)	Traditional treatment group (mean ± SD)	P-value
Baseline	7.8 ± 1.2	7.6 ± 1.1	-
3 months	2.5 ± 1.5	3.8 ± 1.2	<0.001
6 months	1.2 ± 0.9	4.5 ± 1.0	<0.001
12 months	0.6 ± 0.5	5.2 ± 0.8	<0.001

At three months, the laser therapy group showed a substantial reduction, with a mean VAS score of 2.5 (±1.5), while the traditional treatment group had a higher score of 3.8 (±1.2). This pattern persisted at six months, with mean VAS scores of 1.2 (±0.9) in the laser therapy group and 4.5 (±1.0) in the traditional treatment group. At the 12-month mark, the laser therapy group exhibited the lowest mean VAS score of 0.6 (±0.5), indicating a sustained reduction in dentin hypersensitivity. Statistical analysis revealed significant differences at all time points (p < 0.001), underscoring the superior efficacy of laser therapy in reducing dentin hypersensitivity compared to traditional treatment.

At baseline, both groups had elevated tactile hypersensitivity scores (4.2 ± 1.5 for the laser therapy group and 4.0 ± 1.3 for the traditional treatment group). After three months, the laser therapy group showed a substantial reduction (1.5 ± 1.0), while the traditional treatment group had a higher score (2.8 ± 1.2). This trend persisted at 6 and 12 months, with the laser therapy group maintaining lower mean scores (0.8 ± 0.7 and 0.4 ± 0.3, respectively) compared to the traditional treatment group (3.5 ± 1.0 and 4.0 ± 0.7, respectively). The differences were statistically significant at all time points (p < 0.001), affirming that laser therapy consistently outperformed traditional treatment in reducing tactile hypersensitivity (Table 3).

**Table 3:** Tactile hypersensitivity assessments. A p-value <0.05 was considered significant. SD: standard deviation

Time point	Laser therapy group (mean ± SD)	Traditional treatment group (mean ± SD)	P-value
Baseline	4.2 ± 1.5	4.0 ± 1.3	-
3 months	1.5 ± 1.0	2.8 ± 1.2	<0.001
6 months	0.8 ± 0.7	3.5 ± 1.0	<0.001
12 months	0.4 ± 0.3	4.0 ± 0.7	<0.001

Table 4 provides insights into the adverse events. In the laser therapy group, five participants reported mild discomfort, and two reported temporary tooth sensitivity. Conversely, the traditional treatment group had eight reports of mild discomfort and five of temporary tooth sensitivity. Importantly, the majority of the participants in both groups (62 in the laser therapy group and 56 in the traditional treatment group) experienced no adverse events. This demonstrates a favorable safety profile for both interventions, with a slightly lower incidence of adverse events in the laser therapy group.

**Table 4:** Adverse events. n: number of participants.

Adverse event	Laser therapy group (n = 69)	Traditional treatment group (n = 69)
Mild discomfort	5	8
Temporary tooth sensitivity	2	5
No adverse events	62	56

## Discussion

Dentin hypersensitivity, characterized by sharp, transient pain in response to various stimuli, poses a significant challenge in dental practice [12]. While conventional desensitizing treatments have been the mainstay, emerging modalities such as laser therapy have garnered attention for their potential efficacy [7,8].

The study’s primary outcomes, as measured by VAS scores, consistently demonstrated the superior efficacy of laser therapy in reducing dentin hypersensitivity. At 3, 6, and 12 months, the laser therapy group exhibited significantly lower VAS scores than the traditional treatment group. This aligns with previous studies indicating that laser therapy can modulate neural responses, desensitize nerve endings, and promote dentin-tubule occlusion [6,13]. The sustained reduction in VAS scores confirms the fact that laser therapy may offer a lasting solution for patients with dentin hypersensitivity [14].

Tactile hypersensitivity assessments further supported the superiority of laser therapy. Standardized tactile stimuli consistently elicited lower responses in the laser therapy group than in the traditional treatment group. This aligns with the existing literature suggesting that lasers can influence the neural perception of tactile stimuli, potentially reducing hypersensitivity [15-17]. The comprehensive nature of the assessments, combining subjective and objective measures strengthened the credibility of the findings. An essential aspect of any dental intervention is the safety profile. The results indicated that laser therapy was associated with fewer adverse events, primarily mild discomfort and temporary tooth sensitivity, compared to traditional treatment. Importantly, the majority of the participants in both groups reported no adverse events. This aligns with previous studies that highlight the safety of laser therapy when administered by trained professionals [18-20]. The minimal adverse events observed in the laser therapy group suggest that it can be a well-tolerated and safe option for dentin hypersensitivity management.

The adverse events observed in both groups were consistent with the known side effects of dental procedures. Meticulous reporting and categorization of adverse events contribute to a comprehensive understanding of the safety of interventions. Future research should explore the long-term safety of laser therapy and its potential impact on oral health beyond dentin hypersensitivity management.

The findings of this study have significant implications for clinical practice. Laser therapy has emerged as a promising alternative or adjunct to traditional desensitizing treatments. Its efficacy, as demonstrated by reductions in VAS and tactile hypersensitivity scores, suggests that it could address the unmet needs of patients with persistent dentin hypersensitivity. Dental practitioners should consider incorporating laser therapy into their armamentarium to manage this challenging condition. Moreover, the observed safety profile of laser therapy was encouraging. As patients increasingly seek minimally invasive and comfortable dental procedures, the potential for reduced adverse events associated with laser therapy could enhance patient satisfaction and compliance. However, clinicians must remain vigilant and adhere to safety guidelines considering individual patient characteristics and contraindications.

Although this study contributes valuable insights, it is essential to acknowledge certain limitations that may influence the interpretation of the findings. One notable limitation was the relatively short duration of the study, which was confined to 12 months. A more extended follow-up period would be beneficial to elucidate the long-term efficacy and safety of laser therapy and address concerns about the sustainability of its effects over an extended timeframe.

Furthermore, future research endeavors could investigate the broader impact of laser therapy on patients’ quality of life and oral health-related behaviors. Expanding the scope to include these aspects would offer a more comprehensive and holistic understanding of the clinical utility of laser therapy, beyond its immediate effects on dentin hypersensitivity. Such investigations could provide valuable information on how laser therapy is integrated into patients’ overall well-being and oral healthcare practices, thereby enriching the depth of knowledge in this domain.

## Conclusions

This study comparing laser therapy to traditional treatment for dentin hypersensitivity presents compelling evidence for the efficacy and safety of laser therapy. The consistent reduction in VAS and tactile hypersensitivity scores, coupled with a favorable adverse event profile, suggests that laser therapy is a promising intervention. Dental practitioners should cautiously embrace this modality considering its potential to address the persistent challenge of dentin hypersensitivity. Future research should focus on validating these findings through robust clinical trials and exploring their long-term effects and patient-centered outcomes.
